# Supplementary figures and images for: The adaptor protein TASL is required for age-related B cell emergence and lupus-like disease development in mice
Source: PLoS Biol. 2026 Mar 5;24(3):e3003342. doi: 10.1371/journal.pbio.3003342 (PMC12974799; doi:10.1371/journal.pbio.3003342)

## Supplementary Figure 1

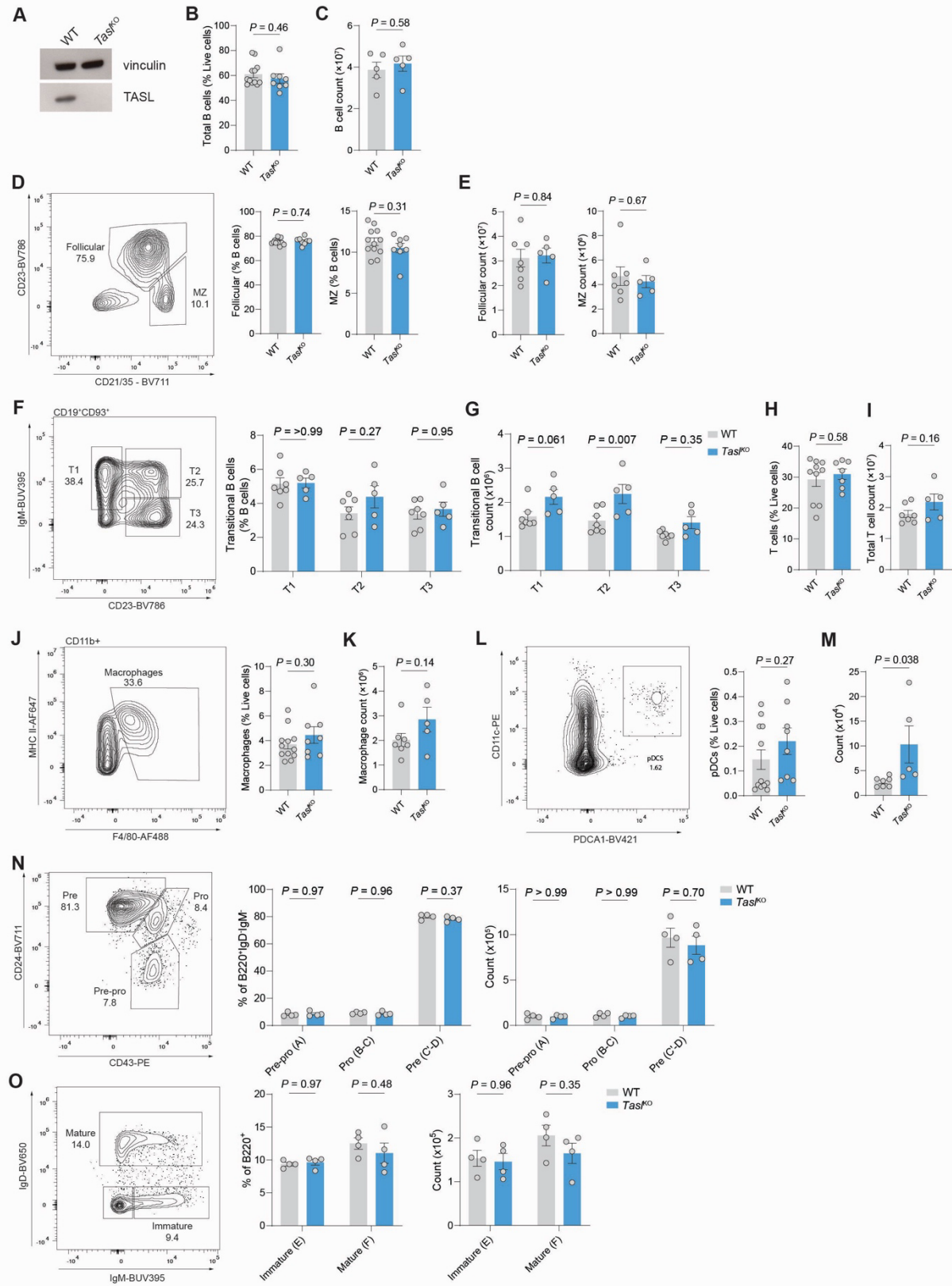

Supplement: S1 Fig — A. Immunoblot of TASL in WT and Tasl KO splenocytes. Vinculin is used as a loading control. Representative of 2 independent experiments. B. Flow cytometric quantification of frequency of live CD19+ B cells in spleens of WT (n = 10) and Tasl KO (n = 8) mice. Data representative of and pooled from 3 independent experiments. C. Count of live CD19+ B cells in spleens of WT (n = 5) and Tasl KO (n = 5) mice. Data representative of and pooled from 2 independent experiments. D. Representative gating and quantification of frequency of CD19+CD23+CD21int follicular B cells and CD19+CD23−CD21+ marginal zone (MZ) B cells in spleens of WT (n = 12) and Tasl KO (n = 8) B cells. Data representative of and pooled from 3 independent experiments. E. Count of follicular B cells and MZ B cells, gated as in D, in spleens of WT (n = 7) and Tasl KO (n = 5) mice. Data representative and pooled from 2 independent experiments. F. Representative gating and quantification of frequency of CD19+CD93+ T1 (CD23-IgM+), T2 (CD23+IgM+), and T3 (CD23+IgM−) B cells in spleens of WT (n = 12) and Tasl KO (n = 8) mice. Data representative of and pooled from 3 independent experiments. G. Count of transitional B cell populations, gated as in F, in spleens of WT (n = 7) and Tasl KO (n = 5) mice. Data representative of and pooled from 2 independent experiments. H. Quantification of total TCRβ+ T cell frequency in spleens of WT (n = 10) and Tasl KO (n = 7) mice. Data representative of and pooled from 3 independent experiments. I. Quantification of total TCRβ+ T cell count in spleens of WT (n = 7) and Tasl KO (n = 5) mice. Data representative of and pooled from 2 independent experiments. J. Representative gating and quantification of frequency and count of splenic CD11b+F4/80+ macrophages in spleens of WT (n = 12) and Tasl KO (n = 8) mice. Data representative of and pooled from 3 independent experiments. K. Count of macrophages, as gated as in F, in spleens of WT (n = 7) and Tasl KO (n = 5) mice. Data representat [file pbio.3003342.s001.pdf]

**Supplementary Figure 2**

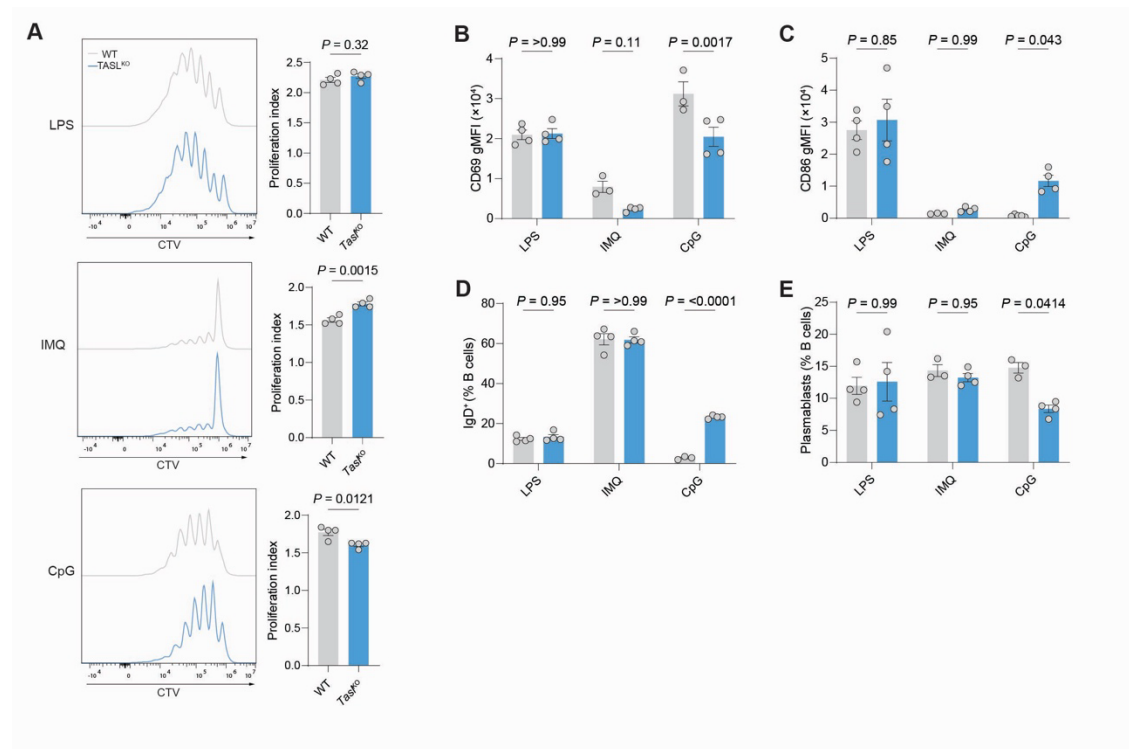

Supplement: S2 Fig — A. Representative histogram and proliferation index of isolated splenic B cells from WT or Tasl KO mice (n = 4) stimulated for 72 hour with either lipopolysaccharide (LPS), imiquimod (IMQ), or CpG. Data representative of 2 independent experiments. B. Flow cytometric measurement of CD69 gMFI in isolated splenic B cells from either WT (n = 4) or Tasl KO (n = 4) male mice after stimulation with either LPS, IMQ, or CpG for 24 hour. Representative of two independent experiments. C. Flow cytometric measurement of CD86 gMFI in isolated splenic B cells from either WT (n = 4) or Tasl KO (n = 4) male mice after stimulation with either LPS, IMQ, or CpG for 24 hour. Representative of two independent experiments. D. Quantification of IgD+ B cells as percentage of total B cells, after stimulation of isolated splenic B cells from male WT (n = 4) or Tasl KO (n = 4) mice with either LPS, IMQ, or CpG for 72 hour. Representative of 2 independent experiments. E. Quantification of plasmablasts as CD19+IgD−CD138+IRF4+ B cells after culture of male B cells with either LPS, IMQ, or CpG for 72 hour (n = 4). Representative of 2 independent experiments. Statistical significance was calculated using two-tailed unpaired t test (A, G) or two-way ANOVA with Šidák’s multiple testing correction (B–E). Data are presented as mean ± S.E.M. Each point represents a single mouse. The data underlying this figure can be found at: DOI https://doi.org/10.5281/zenodo.18649676 (PDF) [file pbio.3003342.s002.pdf]

## Supplementary Figure 3

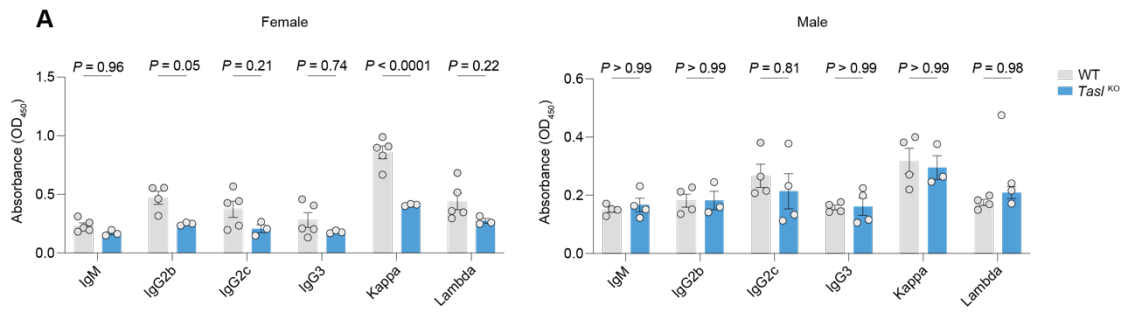

Supplement: S3 Fig — A. Immunoglobulin isotyping of unimmunized female WT (n = 5) or Tasl KO (n = 3) or male WT (n = 4) or Tasl KO (n = 4) mice. Data are representative of 2 independent experiments. Statistical significance calculated by two-way ANOVA with Šidák’s multiple testing correction. Data are presented as mean ± S.E.M. Each point represents a single mouse. The data underlying this figure can be found at: DOI https://doi.org/10.5281/zenodo.18649676 (PDF) [file pbio.3003342.s003.pdf]

## Supplementary Figure 4

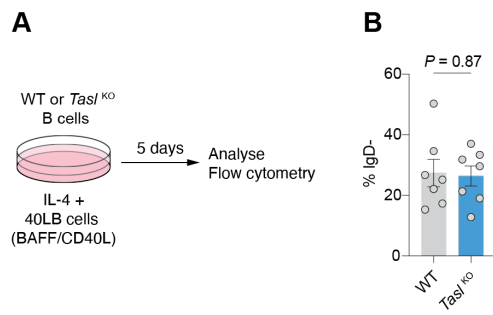

Supplement: S4 Fig — A. Schematic of iGB culture system. Isolated naïve splenic B cells from WT (n = 7) or Tasl KO (n = 7) mice were seeded on a layer of irradiated fibroblasts expressing BAFF and CD40L, with IL-4 and incubated for 5 days. B. Quantification of CD38-GL-7+ iGCB cells from (F). Statistical significance was calculated using two-tailed unpaired t test. Data are presented as mean ± S.E.M. Each point represents a single mouse. The data underlying this figure can be found at: DOI https://doi.org/10.5281/zenodo.18649676S (PDF) [file pbio.3003342.s004.pdf]

## Supplementary Figure 5

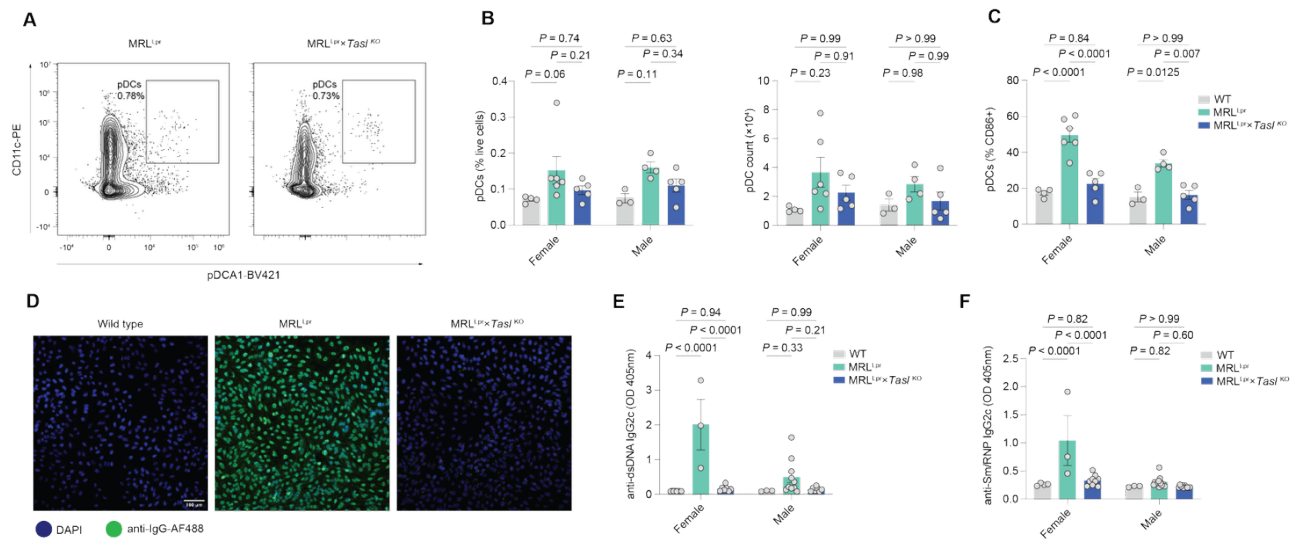

Supplement: S5 Fig — A. Representative flow cytometric gating of CD19−TCRb−CD11c+pDCA1+ pDCs in MRLLpr and MRLLpr ×Tasl KO mice. B. Quantification of pDC proportion and count in 20-week old female WT (n = 4), MRLLpr (n = 6), and MRLLpr ×Tasl KO (n = 5) mice, and male WT (n = 3), MRLLpr (n = 4), and MRLLpr ×Tasl KO (n = 5) mice. Data representative of and pooled from 2 independent experiments. C. Quantification of CD86hi pDCs in 20-week old female WT (n = 4), MRLLpr (n = 6), and MRLLpr ×Tasl KO (n = 5) mice, and male WT (n = 3), MRLLpr (n = 4), and MRLLpr ×Tasl KO (n = 5) mice. Data representative of and pooled from 2 independent experiments. D. Representative images of anti-nuclear antibodies (ANAs) in serum of 20-week old WT, MRLLpr and MRLLpr ×Tasl KO mice, detected by immunofluorescence. Scale bar represents 100μm. E. ELISA quantification of anti-dsDNA IgG2c in sera of 20-week old female WT (n = 4), MRLLpr (n = 3), and MRLLpr ×Tasl KO (n = 7) mice, and male WT (n = 3), MRLLpr (n = 10), and MRLLpr ×Tasl KO (n = 7) mice. Data representative of and pooled from 3 independent experiments. F. ELISA quantification of anti-Sm/RNP IgG2c in sera of 20-week old female WT (n = 4), MRLLpr (n = 3), and MRLLpr ×Tasl KO (n = 9) mice, and male WT (n = 3), MRLLpr (n = 10), and MRLLpr ×Tasl KO (n = 7) mice. Data representative of and pooled from 3 independent experiments. Statistical significance was calculated using two-way ANOVA with Šidák’s multiple testing correction. Data are presented as mean ± S.E.M. Each point represents a single mouse. The data underlying this figure can be found at: DOI https://doi.org/10.5281/zenodo.18649676 (PDF) [file pbio.3003342.s005.pdf]
