# Supplementary material for: The adaptor protein TASL is required for age-related B cell emergence and lupus-like disease development in mice
Source: PLoS Biol. 2026 Mar 5;24(3):e3003342. doi: 10.1371/journal.pbio.3003342 (PMC12974799; doi:10.1371/journal.pbio.3003342)

Blot 1 –

Immunoblot of TASL in WT and *Tasl* ^{KO} splenocytes. Vinculin is used as a loading control.

Blots were scanned using a Canon scanner.

Loaded as: ladder WT KO

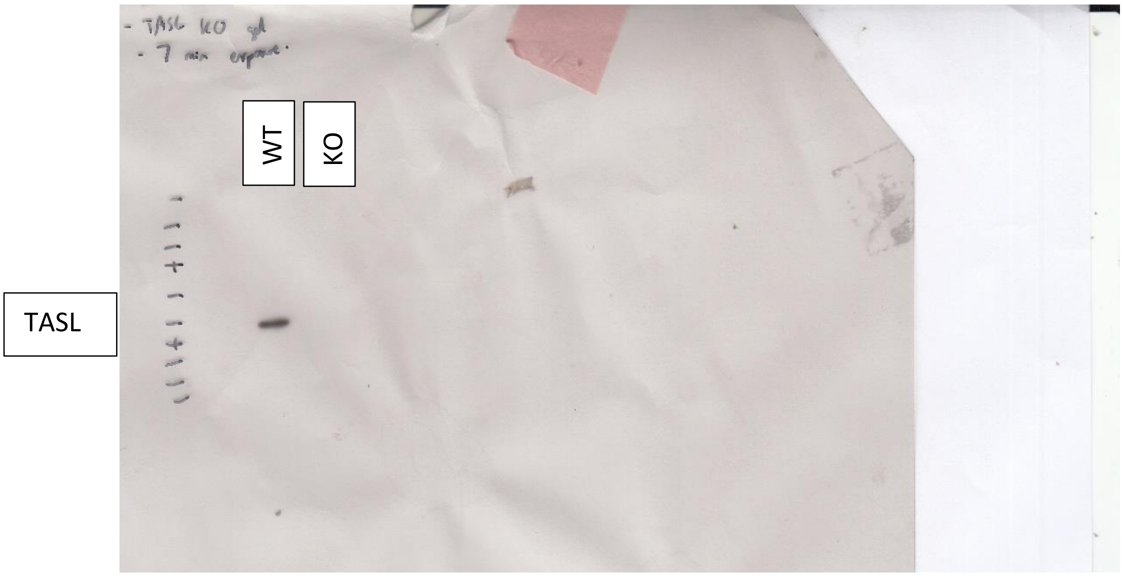

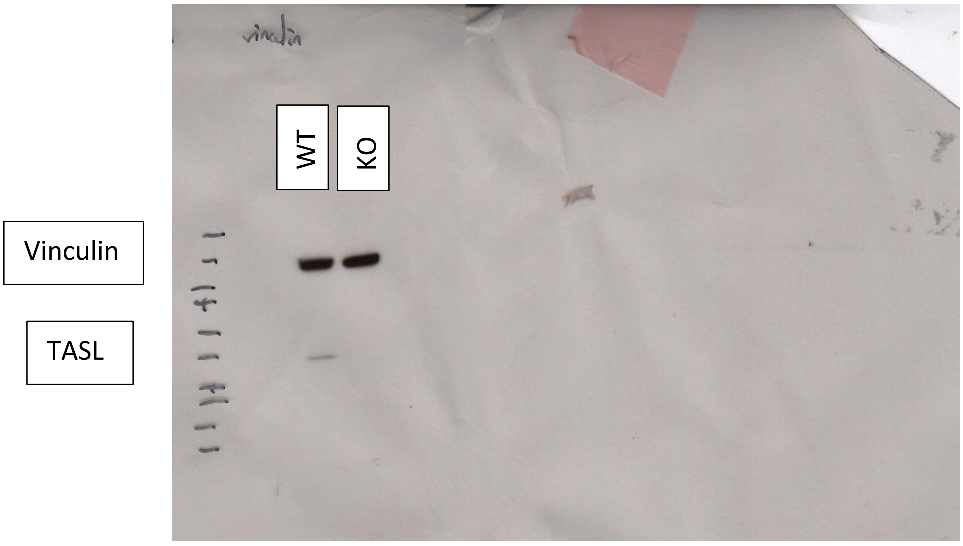

Blot 2 - Repeat

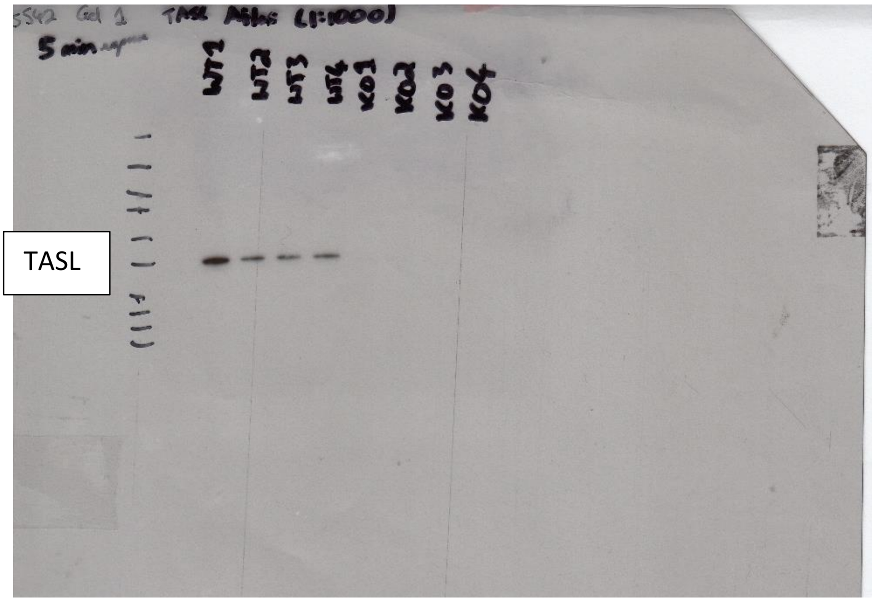

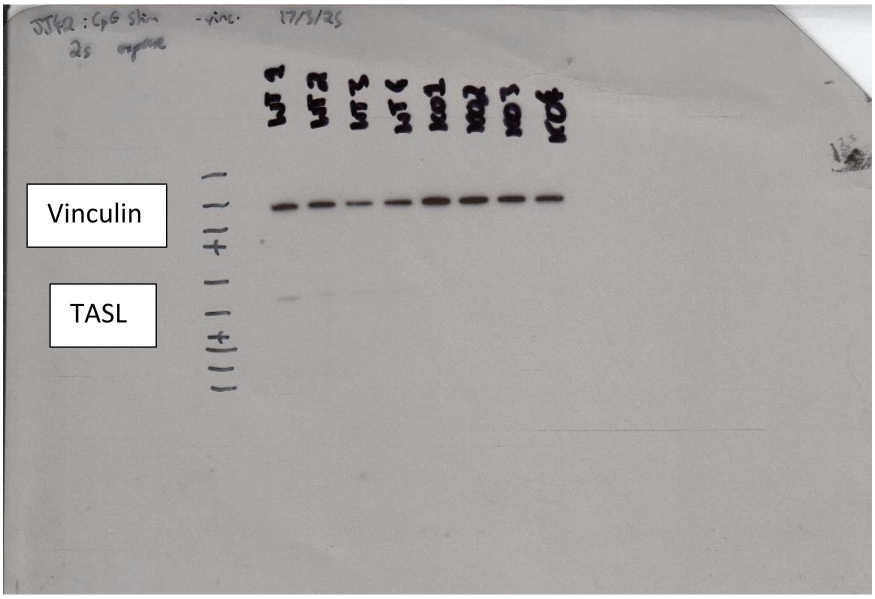

Supplement: S1 Raw Images — (PDF) [file pbio.3003342.s006.pdf]
