# Supplementary material for: The adaptor protein TASL is required for age-related B cell emergence and lupus-like disease development in mice
Source: PLoS Biol. 2026 Mar 5;24(3):e3003342. doi: 10.1371/journal.pbio.3003342 (PMC12974799; doi:10.1371/journal.pbio.3003342)
Supplement: Attachment — Submitted filename: TASL response to reviewers.pdf [file pbio.3003342.s009.pdf]

Reviewer #1:

TASL is an adaptor in the IRF5 signaling pathway and is involved in the phosphorylation of IRF5 in response to TLR7 or TLR9 stimulation. TASL has been shown to play a role in the activation and function of B cells, and in the development of autoimmunity in chemically induced models of lupus. The current manuscript by Johnstone et al. delineates the role of TASL in humoral and autoimmune B cell responses in the extrafollicular age-associated B cell (ABC) and follicular GC differentiation pathways in the context of foreign antigen and spontaneous autoimmunity. The manuscript is novel and significant in highlighting the role of TASL in GC and ABC responses in both foreign antigen driven and spontaneous lupus-like autoimmune responses. I have the following comments and addressing the comments will significantly improve the manuscript.

(1) The extrafollicular ABC and follicular GC responses have been implicated in both protection and in autoimmunity. In the current manuscript, the authors used NP-CGG as a model antigen to determine the role of TASL in protective humoral responses via the ABC and GC pathways. The authors found reduced GC and plasmablast (PB) responses in TASL KO mice compared to wild type control mice. They did not find any difference in affinity maturation and anti-NP IgG1 (Th2) response, but anti-NP IgG2c (Th1) response was reduced. They then went on to show no difference in GC response between wild type and TASL KO GC responses in a competitive bone marrow chimera environment. From these data the authors concluded that TASL is primarily required for the extrafollicular response, with a limited role in the GC reaction. In a competitive chimera, was there any difference in plasmablast response as this was not
shown?

We thank the reviewer for their positive comments and experimental suggestions. As the chimera experiment originally shown in Fig. 2g used immunisation with SRBC, a T-dependent antigen, it is not ideal for assessing the extrafollicular response (EF), and indeed *Tasl* $^{KO}$ plasmablasts were not outcompeted (Reviewer Fig 1a, new Fig. 2h). To properly address the reviewer's question, we have now repeated the chimera experiment in the same format, but instead using immunisation with NP-Ficoll. Here, we saw marked out-competition of *Tasl* $^{KO}$ plasmablasts, which was concordant with the reduction in anti-NP IgM reported in the original Figure 2h (added to Fig 2j; Reviewer Fig 1b). Altogether these chimera experiments place the defect in humoral immunity seen with loss of TASL at the earliest stages of B cell activation, confirming that the EF response is most impacted.

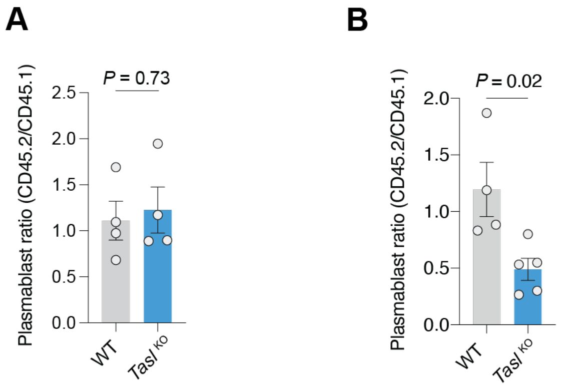


Reviewer Fig 1. A. Ratio of CD45.2/CD45.1 plasmablasts following immunisation with SRBC (data from Fig 2). B. Ratio of CD45.2/CD45.1 plasmablasts following immunisation with NP-Ficoll (new experiment).

Also, was there any difference in anti-SRBC IgG1 and IgG2c responses? The authors should also measure NP-specific GC and plasmablast responses as total GC and PB responses constitute both immunization-induced and spontaneous GC B cell and PB responses.

We focused on assessing antibody responses to NP, as this is a very well-established and reproducible T-dependent antigen with reliable reagents. We used quantification of anti-NP antibodies of various isotypes and assessed affinity maturation (Fig. 2e-f). We find negligible spontaneous GC formation (<0.1% of B cells) in unimmunised mice in our animal facility, and there are no detectable anti-NP antibodies. We therefore do not think this will affect our overall conclusions.

(2) In Figure 3A-C, the authors determined spontaneous ABC formation in 8-month-old wild type and TASL KO mice without examining GC formation at this age. In Figure 3D-F, they differentiate B cells into ABCs through in vitro stimulation with R848, IL-21, anti-CD40 and anti-IgM. These stimuli also differentiate B cells into GC-like cells and authors could demonstrate whether GC-like B cell responses were equally reduced in the absence of TASL. Both steady-state GC and ABC responses could generate IgG2c Abs.

We thank the reviewer for this suggestion. We did not specifically examine GC-like B cell formation in our *in vitro* ABC differentiation experiments. We do think this is an important point, and to further complement our *in vivo* data, we have generated GC-like B cells using a dedicated protocol (iGB system)[1]. In this approach, B cells are cultured on a fibroblast layer expressing CD40L and BAFF, in the presence of IL-4. This leads to GC-like B cells, and is a widely established experimental system. We did not detect a difference in GC-like B cell formation. The data are shown below, and have been added to Supplementary Fig. 2f-g.

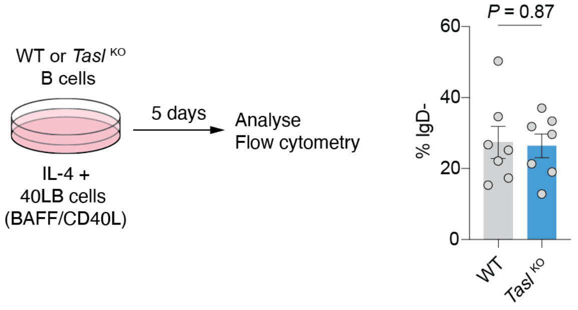


Reviewer Fig 2. iGB system and iGCBC formation in WT and *Tasl KO* B cells.

(3) Plasmablast, ABC and GC responses are all equally reduced in lupus-prone B6.MRLlpr mice and therefore arguing for ABC alone is not well supported.

Our intention in the manuscript is not to claim that ABCs are uniquely reduced in $MRL^{Lpr}$ × *Tasl* $^{KO}$ mice, and to the best of our knowledge we have not done so.

(4) Showing immunofluorescent imaging of GC reaction in lupus-prone mice would reveal whether GC formation is affected in TASL KO mice which will strengthen the manuscript.

We agree that this would be a useful addition, and have now imaged spleen sections from $MRL^{Lpr}$ and $MRL^{Lpr}$ × *Tasl* $^{KO}$ mice (Reviewer Fig. 3), and added to Fig. 4d.

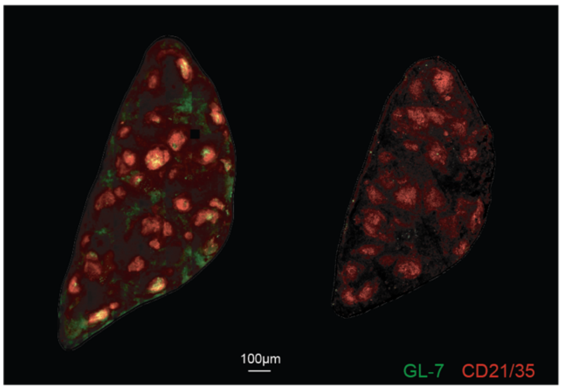


Reviewer Fig. 3. Representative immunofluorescence images of spleens of 20 week old MRL$^{Lpr}$ and MRL$^{Lpr}$ × *Tasl* $^{KO}$ mice. GCs are indicated by GL-7-AF488, and follicles by anti-CD21/35-AF647. The scale bar indicates 100µm.

(5) The role of TASL in autoimmunity development was previously shown in chemically induced lupus model. Therefore, to increase the significance and novelty of this manuscript, the authors should investigate the B cell-intrinsic role of TASL using the BM chimera approach, especially the authors already performed BM chimera experiments in this manuscript.

Please see experiments above, added to Fig. 2.

Reviewer #2 (Betsy Barnes):

The short report by Johnstone et al. is a natural extension of the literature on TASL and IRF5, presenting expected results that indicate Taslko and Irf5ko mice phenocopy each other regarding B cell phenotypes and protection from lupus (PMCID: PMC11760370). As such, it is not clear to this Reviewer that the scientific findings, while strong and expected, fulfill the requirements of the journal - Short Reports should be novel, provocative and of general interest, in such a way as to spur future research. Additional comments are listed below.

We thank the reviewer for highlighting the strength of our findings, and for their helpful suggestions. In terms of novelty, whilst we would highlight that this work was submitted under PLOS Biology's scoop protection policy (6 month window), nonetheless we believe that our manuscript significantly contributes to the understanding of ABC biology in general and the role of TASL in autoimmunity specifically, providing a rationale for future work in an important area of immunology.

1. Suppl. Fig1, a significant increase in T2 cells and pDC numbers from Taslko mice are shown (panels G and M). While relatively small, does this phenotype overlap with Irf5ko mice? Authors should discuss.

We appreciate the reviewer's observation. Irf5$^{-/-}$ mice have normal pDC and T2 B cell development[2–4]. We have added a point in the discussion on this.

2. Fig. 1, why the discrepancy in CD69 and CD86 expression in response to CpG stimulation? This is interesting but should be discussed. Also to this point, why do the authors think most effects are seen downstream of CpG rather than IMQ?

We thank the reviewer for pointing this out, and agree that it is an interesting observation which requires further discussion. We are not certain why this discrepancy is seen, but it may reflect differences in the kinetics of CD86 expression *in vitro*, perhaps due to basal differences in signalling. The literature on *IRF5* deletion shows that in most reports, CD86 expression on B cells is reduced following in vitro activation[5,6]. It has been reported in an older work that *Irf5*$^{-/-}$ B cells have higher CD86 expression after 4 days of stimulation, but whether this can be compared to our data is unclear[7]. The differences observed between CpG and IMQ most likely arose because with the dose of IMQ we used, a weaker stimulus is provided than that from CpG.

3. Data from male mice in Fig. 2d and f is curious since there appears to be no change in plasmablast percents and numbers yet there is a significant decrease in IgG2c. Could the authors please discuss this discrepancy? Is there a secretion defect? The authors may need to examine intracellular levels and ELISPOT would be more rigorous to examine on a per cell basis.

We found that there was no difference in the levels of anti-NP IgG1 between mice of either sex or genotype, but a marked reduction in anti-NP IgG2c in all *Tasl* $^{KO}$ mice. Across our work, female mice tended towards a stronger phenotype for most measures, for example resting immunoglobulin levels (Supplementary Figure 3A, reproduced below).

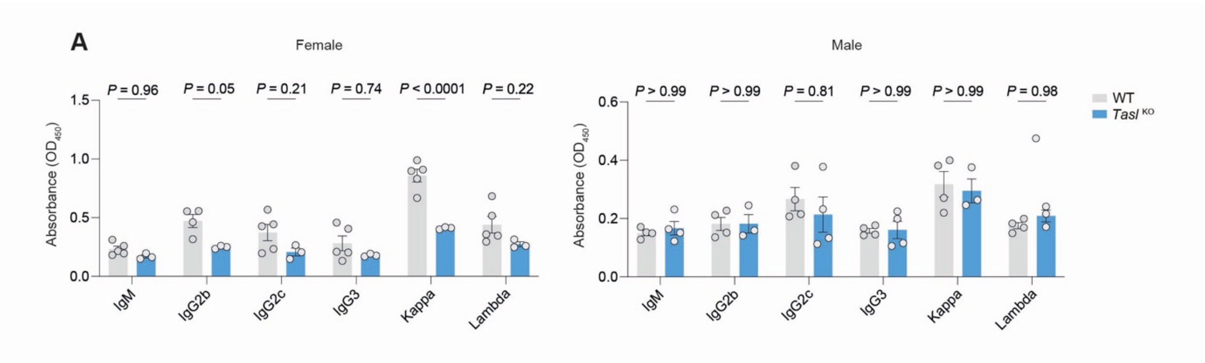


Reviewer Fig. 4

We thank the reviewer for suggesting an ELISPOT assay, which we have now performed (data shown below, and added to Fig. 4g), using immunised male mice. This confirms our findings in serum.

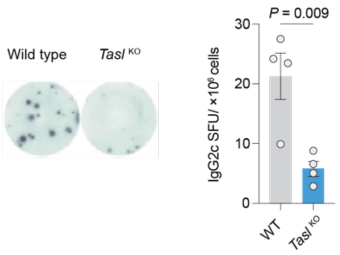


Reviewer Fig. 5

4. Suppl Fig3a, it is very curious to see reduced levels of IgGs at steady state, which needs to be discussed.  Is this similar to Irf5ko mice?

Although this phenotype is not widely reported in the literature to the best of our knowledge, we found two reports of resting immunoglobulin levels in *IRF5* KO mice. In one, there was a modest reduction in most IgG subclasses, which increased in extent with age, but in another no clear difference[7,8]. We were unable to find a description of the sex of the mice in either publication. Due to the potential for the presence of a mutation in *Dock2* in the earlier work, we are unclear as to how this should be interpreted. In our experiments we analysed male and female mice separately, and the relatively mild reduction we observed was only seen in the latter. We have added a section in the discussion around this topic.

5. Fig 2H, what affect does NP-Ficoll have on the generation of PBs, given the conserved decrease in NP-IgM in both male and female Taslko mice?

We found that loss of TASL reduced plasmablast generation following NP-Ficoll immunisation in a similarly sexual dimorphic manner to that seen with SRBCs.

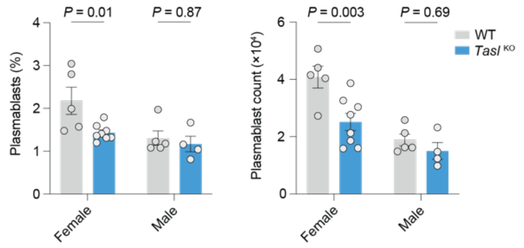


Reviewer Fig. 6

6. Similar to comment 2 above, the authors conclude that Tasl is required for effective transduction of endolysosomal signaling yet only show compelling data for TLR9, with minimal effects in response to TLR7 or TLR4 signaling. This is confusing and is not supported by the data shown.

Whilst TLR4 is not an endosomal TLR, and accordingly despite strong activation there is no difference in phenotype following TASL deletion, unlike the endosomal TLR9, we agree that the TLR7 results show comparatively less difference. We have therefore toned down the subheading.

7. The authors need to discuss similarities and differences between their findings and findings in Irf5ko since the pathways overlap and similar experiments have been performed on Irf5ko mice (PMID: 39572974). This is particularly relevant since the authors end the discussion with a conclusion regarding TLR7-IRF5 signaling while the data support little effect of Tasl in response to the TLR7 ligand IMQ.

We hope that in addressing the reviewers preceding points, we have substantially increased our discussion of the overlap with *Irf5* KO.

8. Some of the representative flow plots do not match data in bar graph. For example, Fig1c shows 92% IgD+ cells from Taslko while the bar graph in panel d shows 50-60%. The same is true for Fig2c and d; 3.1% plasmablasts are shown in Taslko mice while the bar graph shows all values to be below 2%.

We thank the reviewer for picking this up, and apologise. These have now been corrected.

------------------------------------

Reviewer #3:

In the study by Johnstone et al., the data presented in the manuscript adds to the growing evidence that TASL and altered endosomal signalling is a key pathogenic mechanism in SLE by modulating B cell activation. The data are well presented and conducted, and the study will be a robust addition to the field as a short report. A few minor clarifications are needed prior to publication:

We are grateful to the reviewer for their positive view of manuscript and helpful comments.

1 - Figure 1 is the only figure that does not have data from both sexes (female only), do the authors see sex differences on the impact of TASL KO on CpG/TLR stim?

We would kindly direct the reviewer to the data in Supplementary Fig. 2, in which most of the readouts presented in Fig. 1 are also shown in male mice. We did not detect meaningful sexual dimorphism in our *in vitro* experiments.

2 - Please add male and female labels on the graphs themselves rather than just in the legend for Figure 3 for ease of interpretation.

We agree that these additional labels would make data interpretation easier, and have now added them to Fig. 3.

3 - I suggest to combine the two section in the manuscript focused on mrl.lpr data into one as they are currently very short.

Whilst we do agree that these sections are short in terms of text, their respective figures are quite large with numerous panels, and we are concerned that if we combined these then they would be cluttered and distracting.

4 - Please add to the legend the sex of the representative images in Figure 4a.

We apologise for the omission of this label, the representative images in Fig. 4a are from female mice and we have now added this information to the legend.

5 - As the authors importantly highlight in the discussion, that due to the challenges they are using global TASL KO rather than conditional system and therefore in vivo effects could be driven (or at least) partially by changes in myeloid compartment (pDC). Do they authors see changes in this subset including IFN signalling in the lupus model? This may be out of scope

for a short report, but it should at least be added as a limitation of study that they have not enumerated/phenotyped the myeloid compartment in these experiments.

We are grateful to the reviewer for this point. We do indeed have data on pDCs from our MRL$^{Lpr}$ × *Tasl* $^{KO}$ mice experiments, which we have added to Fig. 4k-m.

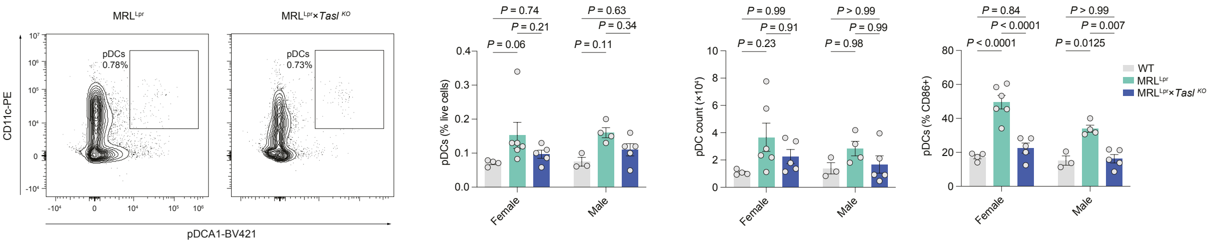


Reviewer Fig. 7

## References


1. Nojima, T. *et al.* In-vitro derived germinal centre B cells differentially generate memory B or plasma cells in vivo. *Nat Commun* **2**, 465–11 (2011).
2. Yasuda, K. *et al.* Phenotype and function of B cells and dendritic cells from interferon regulatory factor 5-deficient mice with and without a mutation in DOCK2. *Int Immunol* **25**, 295–306 (2013).
3. Matta, B., Battaglia, J., Lapan, M., Sharma, V. & Barnes, B. J. IRF5 Controls Plasma Cell Generation and Antibody Production via Distinct Mechanisms Depending on the Antigenic Trigger. *Immunology* **174**, 226–238 (2025).
4. Purtha, W. E., Swiecki, M., Colonna, M., Diamond, M. S. & Bhattacharya, D. Spontaneous mutation of the Dock2 gene in Irf5–/– mice complicates interpretation of type I interferon production and antibody responses. *Proceedings of the National Academy of Sciences* **109**, E898–E904 (2012).
5. Pellerin, A. *et al.* Monoallelic *IRF5* deficiency in B cells prevents murine lupus. *JCI Insight* **6**, (2021).
6. De, S. *et al.* B Cell-Intrinsic Role for IRF5 in TLR9/BCR-Induced Human B Cell Activation, Proliferation, and Plasmablast Differentiation. *Front. Immunol.* **8**, (2018).
7. Savitsky, D. A., Yanai, H., Tamura, T., Taniguchi, T. & Honda, K. Contribution of IRF5 in B cells to the development of murine SLE-like disease through its transcriptional control of the IgG2a locus. *Proceedings of the National Academy of Sciences* **107**, 10154–10159 (2010).
8. Feng, D. *et al.* Irf5-deficient mice are protected from pristane-induced lupus via increased Th2 cytokines and altered IgG class switching. *European Journal of Immunology* **42**, 1477–1487 (2012).
